# Astaxanthin Inhibits JAK/STAT-3 Signaling to Abrogate Cell Proliferation, Invasion and Angiogenesis in a Hamster Model of Oral Cancer

**DOI:** 10.1371/journal.pone.0109114

**Published:** 2014-10-08

**Authors:** J. Kowshik, Abdul Basit Baba, Hemant Giri, G. Deepak Reddy, Madhulika Dixit, Siddavaram Nagini

**Affiliations:** 1 Department of Biochemistry and Biotechnology, Faculty of Science, Annamalai University, Annamalainagar, Tamil Nadu, India; 2 Laboratory of Vascular Biology, Department of Biotechnology, Indian Institute of Technology Madras, Chennai, Tami Nadu, India; 3 Medicinal Chemistry Research Division, Vishnu Institute of Pharmaceutical Education and Research, Narsapur, India; National Cancer Institute, United States of America

## Abstract

Identifying agents that inhibit STAT-3, a cytosolic transcription factor involved in the activation of various genes implicated in tumour progression is a promising strategy for cancer chemoprevention. In the present study, we investigated the effect of dietary astaxanthin on JAK-2/STAT-3 signaling in the 7,12-dimethylbenz[a]anthracene (DMBA)-induced hamster buccal pouch (HBP) carcinogenesis model by examining the mRNA and protein expression of JAK/STAT-3 and its target genes. Quantitative RT-PCR, immunoblotting and immunohistochemical analyses revealed that astaxanthin supplementation inhibits key events in JAK/STAT signaling especially STAT-3 phosphorylation and subsequent nuclear translocation of STAT-3. Furthermore, astaxanthin downregulated the expression of STAT-3 target genes involved in cell proliferation, invasion and angiogenesis, and reduced microvascular density, thereby preventing tumour progression. Molecular docking analysis confirmed inhibitory effects of astaxanthin on STAT signaling and angiogenesis. Cell culture experiments with the endothelial cell line ECV304 substantiated the role of astaxanthin in suppressing angiogenesis. Taken together, our data provide substantial evidence that dietary astaxanthin prevents the development and progression of HBP carcinomas through the inhibition of JAK-2/STAT-3 signaling and its downstream events. Thus, astaxanthin that functions as a potent inhibitor of tumour development and progression by targeting JAK/STAT signaling may be an ideal candidate for cancer chemoprevention.

## Introduction

Signal transducer and activator of transcription 3 (STAT3) protein is a latent cytoplasmic transcription factor that transmits signals from the cell surface to the nucleus when activated by cytokines and growth factors [1]. In particular, interleukin-6 (IL-6) or epidermal growth factor (EGF) stimulate the phosphorylation of STAT3 protein by Janus kinase and activated STAT3 forms a homodimer that translocates to the nucleus where it regulates the expression of genes critical for normal cellular processes such as cell development, differ­entiation, proliferation, survival, angiogenesis, and immune function [2]–[6].

Aberrant activation of JAK/STAT3 signaling has been documented in a wide variety of human tumors, including hematopoietic malignancies and solid tumors such as head and neck, breast, and prostate cancers [7], [8]. Constitutive STAT3 activation contributes to proliferation and oncogenesis by modulating the expression of a variety of genes required for tumor cell survival, proliferation, and angiogenesis, as well as invasion and metastasis and commonly suggests poor prognosis [9]–[11]. Thus, JAK/STAT3 signaling plays a central role in tumorigenesis and is considered an important therapeutic target for novel drug development.

Identification of agents that target STAT3 molecule is likely to be of significance in cancer chemoprevention. Several dietary antioxidants are recognized to block tumour development by targeting the STAT3 signaling network [12]–[15]. Astaxanthin, a non-provitamin A carotenoid predominantly found in microalgae, fungi, plants, sea foods and some birds such as flamingos and quail is a potent antioxidant [16]. Astaxanthin was found to exhibit the highest antioxidant activity among the carotenoids and is widely used in the prevention and treatment of various diseases [17]. AXT has also been demonstrated to exhibit anti-inflammatory and anticancer properties [18], [19].

Recently, we demonstrated that dietary supplementation of AXT induces intrinsic apoptosis by inhibiting PI3/Akt, MAPK, NF-κB and Wnt/β-catenin signaling circuits in the 7,12-dimethylbenz[a]anthracene (DMBA)-induced hamster buccal pouch (HBP) carcinogenesis model [20]. These findings tempted us to hypothesize that AXT that induces apoptosis may block the opposing process of cell proliferation thereby preventing the sequential accumulation of mutations that eventually lead to tumour invasion and angiogenesis. Furthermore, AXT-induced inactivation of the transcription factors NF-κB and β-catenin, central hubs in oncogenic signaling could also impact the JAK/STAT3 pathway. In the present study we demonstrate that dietary AXT inhibits tumour progression based on abrogation of the JAK/STAT3 pathway and its downstream targets cyclin D1, MMP-2, -9, and VEGF in the HBP carcinogenesis model. Furthermore AXT decreased microvascular density, which plays an essential role in tumour development and progression. Cell culture experiments with the endothelial cell line ECV304 were also performed to substantiate the role of astaxanthin in suppressing hypoxia-induced angiogenesis.

## Materials and Methods

### Chemicals

Acrylamide, bovine serum albumin (BSA), bromophenol blue, 7,12-dimethylbenz[a]anthracene (DMBA), hydroxyurea, 2-mercaptoethanol, sodium dodecyl sulphate (SDS) N,N,N′,N′ - tetramethylene diamine (TEMED) and Trizol were purchased from Sigma Chemical Company, St. Louis, MO, USA. Astaxanthin was procured from Bio-Real, Sweden. DMEM-F12 medium, antibiotic solution consisting of penicillin and streptomycin and Alamar blue were from HiMedia Labs, Mumbai, India. Fetal bovine serum of South American origin was from GIBCO, Invitrogen, NY, USA. Power SYBR Green PCR master mix was obtained from Applied Biosystems, California, USA. Antibodies for IL-6, GAPDH, Cyclin D1, PCNA, p21, MMP-2, MMP-9, TIMP-2, RECK, VEGF, VEGFR2, HIF1α, were purchased from Santa Cruz Biotechnology, USA. pJAK-2^tyr1007/1008^, JAK-2, pSTAT-3^tyr705^, STAT-3 and histone (H2B) antibodies and BrdU, STAT-3^tyr705^, total cyclin D1 and pVEGFR2^tyr1175^ ELISA kits were from Cell Signaling Technology, USA. CD-34 antibody was purchased from Novocastra, Germany. Matrigel was from BD Biosciences, USA. All other reagents used were of analytical grade.

### Animals and ethics statement

Eight to ten weeks old male Syrian hamsters weighing between 100–110 g were used in this study. Animals were obtained from Central Animal House, Annamalai University, India. The animals were housed four to a cage and provided with standard pellet diet and water ad libitum. The animal health was monitored daily during the study. The protocols for the animal experiments were approved by the Institutional Animal Ethics Committee, Annamalai University and conducted according to the guidelines laid down by the Committee for the Purpose of Control and Supervision on Experiments on Animals (CPCSEA).

### Treatment schedule

The animals were randomized into experimental and control groups and divided into 4 groups of 5 animals each. In group 1, the right buccal pouches of hamsters were painted with 0.5% DMBA in liquid paraffin three times a week for 14 weeks [21]. Group 2 animals received in addition to DMBA, a basal diet containing 15 mg/kg bw of astaxanthin [20], [22]. Group 3 animals received astaxanthin (15 mg/kg bw) alone for 14 weeks. Group 4 animals received basal diet alone and served as an untreated control. The experiment was terminated at 14 weeks and all animals were sacrificed by cervical dislocation after an overnight fast. The buccal pouch tissues were immediately subdivided and processed for distribution to each experiment.

### Cell culture

ECV 304 cell line was used and cultured in DMEM basal medium with 10% fetal bovine serum with antibiotics. ECV304 is an endothelial cell line derived from human umbilical vein endothelial cells (HUVECs) through spontaneous transformation [23]. Compared to primary HUVEC cultures, use of ECV304 cells has a practical advantage as these cells exhibit enhanced and reproducible capacity for in vitro angiogenesis are thus an ideal choice for cell culture based angiogenesis assays [24]. Confluent cultures of ECV304 cells were subcultured and maintained in CO_2_ incubator at 37°C. For matrigel assay, cells were maintained in DMEM basal medium with 4% fetal bovine serum. For hypoxic condition, cells were incubated in hypoxia chamber with 1% O_2_.

### RNA extraction and quantitative real-time RT-PCR

Total RNA from the buccal pouch tissues was extracted using Trizol reagent as described previously [25]. The RNA concentration was determined from the optical density at a wavelength of 260 nm (using an OD260 unit equivalent to 40 µg/ml of RNA). 5 µg of isolated total RNA was reverse-transcribed to cDNA in a reaction mixture containing 4 µl of 5× reaction buffer, 2 µl of dNTP mixture (10 mM), 20 units of RNase inhibitor, 200 units of avian-myeloblastosis virus (AMV) reverse transcriptase and 0.5 µg of oligo(dT) primer (Promega, WI, USA) in a total volume of 20 µl. The reaction mixture was incubated at 42°C for 60 min and the reaction terminated by heating at 70°C for 10 min. The cDNA was stored at −80°C until further use.

Quantitative RT-PCR was performed using Power SYBR Green master mix according to the manufacturer’s instructions using a StepOne Plus thermocycler (Applied Biosystems). To the 1×PCR master mix, 2.5 µl of each cDNA was added in a 20 µl final volume. The PCR conditions were as follows: 95°C for 5 min, 40 cycles of 30 s at 95°C, 30 s at 52 to 60°C (based on the target), and 60 s at 72°C. Relative quantitative fold change compared to control was calculated using the comparative Ct method, where Ct is the cycle number at which fluorescence first exceeds the threshold. The Ct values from each sample were obtained by subtracting the values for GAPDH Ct from the target gene Ct value. The specificity of resulting PCR products was confirmed by melting curves.

### Western blotting

Proteins were extracted from tissue sample using lysis buffer containing 62.5 mM Tris (pH 6.8), 10% SDS, 5% 2-mercaptoethanol, 10% glycerol and bromophenol blue. Nuclear and cytoplasmic fractions were separated as described by Legrand-Poels et al. [26]. Equal amount of protein extracts were loaded onto SDS-PAGE and the resolved proteins were transferred to polyvinylidene difluoride membranes. The blots were then incubated for 2 h in 1X PBS containing 5% non-fat dry milk. The membranes were then probed with primary and secondary antibodies as per manufacturer’s instructions. The proteins were visualized using enhanced chemiluminescence detection reagents (Sigma). Densitometry was performed on IISP flat bed scanner and quantitated with Total Lab 1.11 software.

### ELISA

The levels of pSTAT^tyr705^, total cyclin D1 and pVEGFR2^tyr1175^ were determined using sandwich ELISA kit (Cell Signaling Technology, USA) according to the manufacturer’s instructions.

### Immunohistochemistry

Paraffin embedded tissue sections were deparaffinised, rehydrated and subjected to antigen retrieval and endogenous peroxidase blocking. Then the sections were incubated with pSTAT-3 rabbit monoclonal, PCNA, MMP-2 and VEGF rabbit polyclonal antibodies at room temperature for 3 h. The slides were washed with TBS and then incubated with biotin-labeled secondary antibody followed by streptavidin–biotin–peroxidase (Dako, Carprinteria, CA, USA) for 30 min each at room temperature. The immunoprecipitate was visualized by treating with 3,3′-diaminobenzidine and counterstaining with hematoxylin. The tissues were then photographed using an Inverted Fluorescent Microscope (Leica Microsystem Vertrieb GmbH, Wetzler, Germany) attached with digital camera DFC295.

### Microvascular density (MVD)

Microvascular density was assessed by immunohistochemical staining with anti-CD34 antibody. The areas of highest neovascularization were located and the images captured in a minimum of five different fields. Microvessels were counted by two independent investigators and the data represented as number of vessels/field of view.

### Molecular docking

Molecular docking was done using Schrodinger suite 2013. Astaxanthin was retrieved from PubChem (www.ncbi.nlm.nih.gov/pccompound) and proteins STAT-3 and VEGF was retrieved from the protein databank with PDB ID: 3CWG and 3V2A respectively. Receptor grid generation and ligand docking were performed by employing Glide Xp docking algorithm.

### Alamar blue assay

Cell viability was measured by Alamar Blue assay [27]. Briefly, Alamar blue (Resazurin sodium salt from Himedia) was dissolved in phosphate buffered saline pH 7.4 to make a stock of 5 mg/mL and a final working concentration of 0.1 mg/mL in cell culture medium. Resazurin is a redox indicator, which measures the reducing environment of the cell by reducing to a highly fluorescent resorufin. Astaxanthin at different concentrations (5, 10, 20, 50, 100, 200 and 400 µM) was added to the cells and after 24 h, Alamar blue dye was added and the plates were incubated at 37°C for 4 h. The color change was monitored colorimetrically at 595 nm and 570 nm to evaluate oxidized versus reduced forms respectively of the reagent by using multi-mode plate reader.

### BrdU assay

Cell proliferation was analysed by measuring DNA synthesis with bromodeoxyuridine (BrdU) enzyme-linked immunosorbent assay (ELISA) kit (Cell Signaling Technology, USA), according to the manufacturer’s instructions. Briefly, 1×10^4^ cells were seeded into a 96-well microplate and cultured with or without astaxanthin (50 µM) for 24 h. The cells were then labelled with BrdU for 6 h. After fixation, the cells were incubated with 100 µl of anti-BrdU antibody for 60 min. After washing, 100 µl of secondary antibody was added and incubated for 30 min, then 100 µl of substrate (tetramethylbenzidine) was added to each well, and the plates were incubated at room temperature for 30 min. The absorbance at 450 nm was measured with an ELISA reader.

### Migration assay

Confluent monolayers of ECV 304 cells in 24 well plates were scratched with a 200 µl pipette tip and incubated in normoxic and hypoxic condition with and without 50 µM astaxanthin. 5 mM hydroxyurea was also added to inhibit cell proliferation. Cells were photographed under a microscope at a magnification of 4x at 0 and 24 hours [28].

### Matrigel tube formation assay

Pre-cooled 96 well plates were coated with 80 µl of matrigel (BD) and incubated at 37°C for half an hour. Equal number of cells (20,000/well) were added in each well with and without hypoxic condition and in presence and absence of astaxanthin (50 µM) and incubated in CO_2_ incubator at 37°C for 14 hours. Images were captured in minimum five different fields and data represented finally as number of tubes/field of view [29].

### Statistical analysis

Statistical analysis was carried out using a nonparametric Mann–Whitney test (Stats Direct, United Kingdom) for *in vivo* and Tukey posthoc test for *in vitro* experiments. A probability value of less than 0.05 was considered significant.

## Results

### Tumor incidence

The tumor incidence has been reported in an earlier study [20]. At the end of the experimental period the tumor incidence was 100% with a mean tumor burden of 82.25 mm^3^ in hamsters painted with DMBA (group 1). Dietary supplementation of astaxanthin (15 mg/kg bw) to DMBA painted hamsters did not induce any gross tumours in the buccal pouch and histological examination revealed only mild hyperplasia. In hamsters fed astaxanthin alone and in control groups, the epithelium was normal, intact, and continuous.

### Astaxanthin inhibits JAK/STAT signaling by restraining the phosphorylation of STAT-3

**Figure 1:**
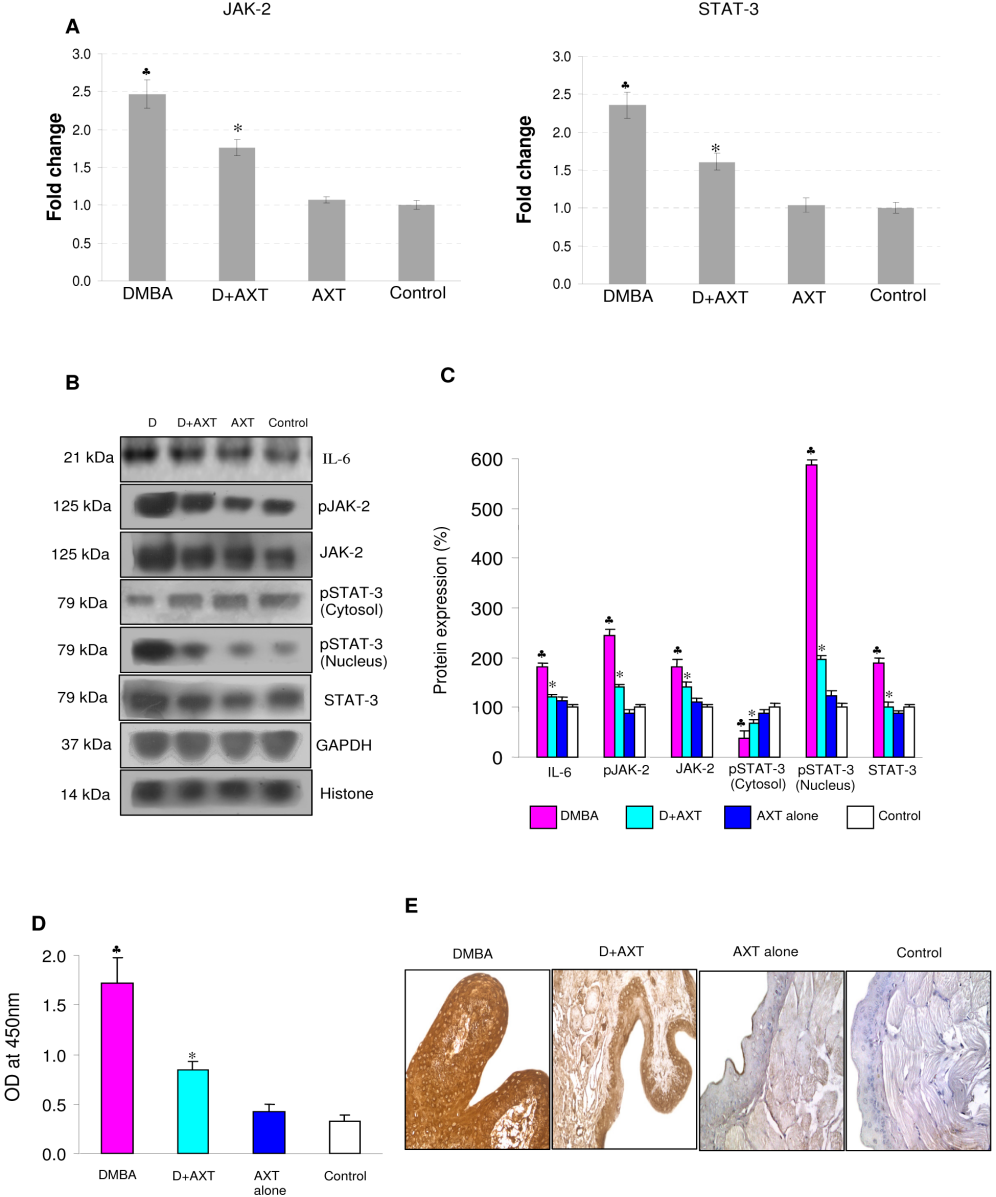
mRNA and protein expression of JAK-2 and STAT-3 in the buccal pouch tissues of experimental and control animals. (mean ± SD; n = 3). A. Transcript expression level of JAK-2 and STAT-3 in various experimental groups as determined by kinetic PCR. Data are the mean ± SD of three independent experiments.^ ♣^p<0.05 versus control. *p<0.05 versus DMBA. B. Representative immunoblot analysis. Protein samples (100 μg/lane) resolved on SDS-PAGE were probed with corresponding antibodies. GAPDH was used as loading control for cytosol and whole tissue homogenates. Histone H2B was used as loading control for nuclear proteins. Phosphorylated proteins are normalized by their unphosphorylated form. C. Densitometric analysis. The protein expression from control lysates for three determinations was designated as 100% in the graph. Each bar represents the protein expression of three determinations.^ ♣^p<0.05 versus control. *p<0.05 versus DMBA. D. Levels of pSTAT^tyr705^ (ELISA). E. Representative photomicrographs of immunohistochemical staining of pSTAT-3 in control and experimental animals (20X).

As STAT 3 is constitutively activated in a wide range of malignancies, we first analyzed the mRNA expression of JAK-2 and STAT-3 by quantitative RT-PCR analysis. Our results revealed that dietary supplementation of astaxanthin significantly reduced the mRNA expression of key molecules involved In JAK/STAT signaling compared to DMBA-painted animals (Figure 1A). No significant differences were observed in animals treated with astaxanthin alone compared to control. Further to explore the mechanism by which astaxanthin regulates JAK/STAT signaling, we next determined the protein expression of IL-6, JAK-2, pJAK-2, STAT-3 and pSTAT-3 by western blotting. We found that topical application of DMBA significantly increased the expressions of these genes compared to control. Simultaneous dietary administration of astaxanthin inhibited JAK/STAT signaling by decreasing the levels of IL-6, JAK/STAT phosphorylated forms and subsequent translocation to the nucleus (Figure 1B & 1C). This was further confirmed by decreased level of pSTAT^tyr705^ (ELISA) in AXT treated group (Figure 1D). Immunohistochemical staining also confirmed that astaxanthin supplementation resulted in significant decrease in the expression of pSTAT-3 compared to DMBA-painted animals (Figure 1E).

### Astaxanthin impedes cell proliferation, invasion and angiogenesis via inhibiting JAK/STAT pathway

**Figure 2:**
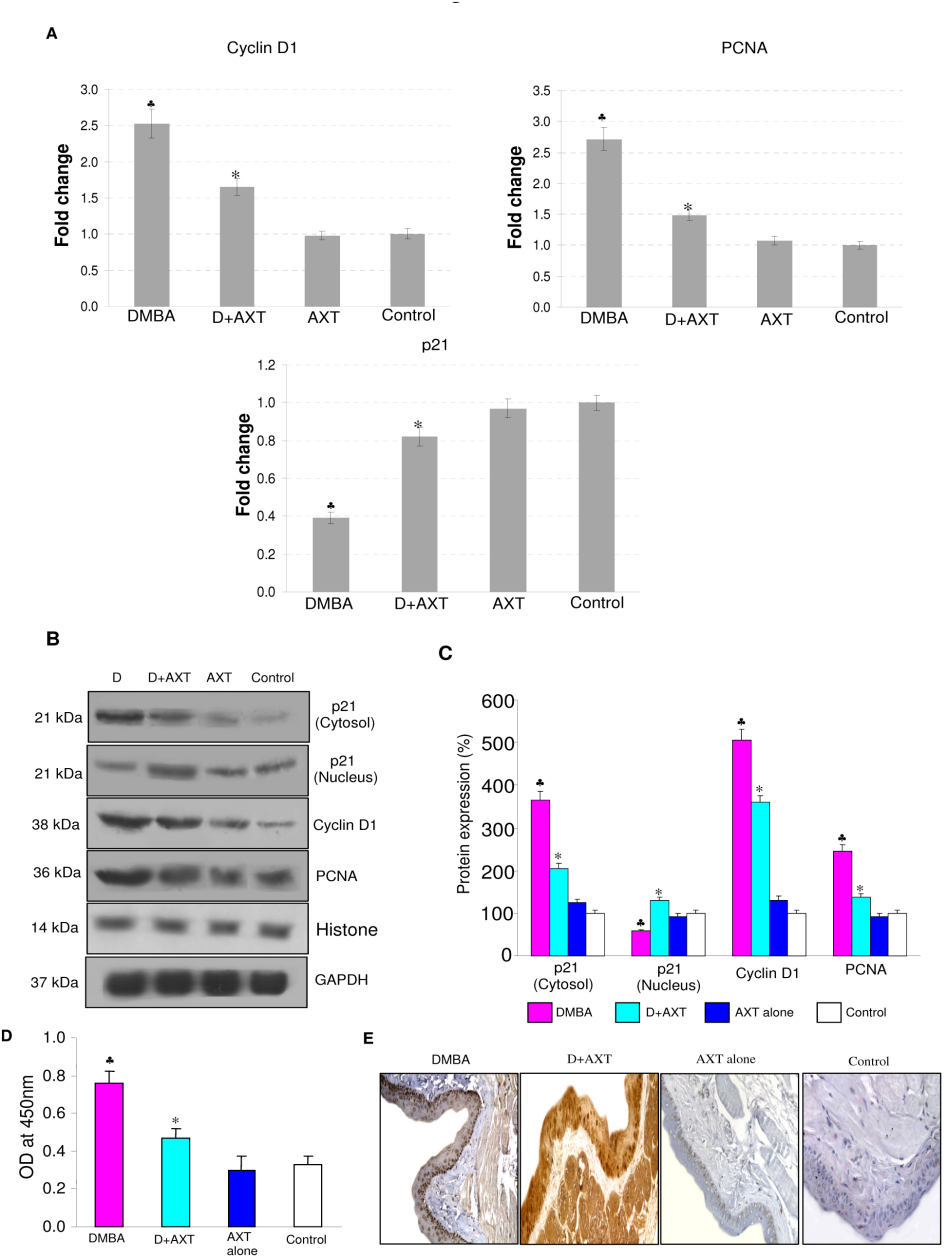
mRNA and protein expression of p21, Cyclin D1 and PCNA in the buccal pouch tissues of experimental and control animals. (mean ± SD; n = 3). A. Transcript expression level of p21, cyclin D1 and PCNA in various experimental groups as determined by kinetic PCR. Data are the mean ± SD of three independent experiments.^ ♣^p<0.05 versus control. *p<0.05 versus DMBA. B. Representative immunoblot analysis. Protein samples (100 μg/lane) resolved on SDS-PAGE were probed with corresponding antibodies. GAPDH was used as loading control for cytosol and whole tissue homogenates. Histone H2B was used as loading control for nuclear proteins. C. Densitometric analysis. The protein expression from control lysates for three determinations was designated as 100% in the graph. Each bar represents the protein expression of three determinations.^ ♣^p<0.05 versus control. p<0.05 versus DMBA. D. Levels of total cyclin D1 (ELISA). E. Representative photomicrographs of immunohistochemical staining of PCNA in control and experimental animals (20X).

As STAT-3 activation regulates the transcription of genes involved in cell proliferation, invasion, and angiogenesis, we next investigated the effect of astaxanthin on markers of cell proliferation. Dietary supplementation of astaxanthin significantly reduced the mRNA and protein expression of cyclin D1 and PCNA. Furthermore, AXT increased nuclear p21 expression relative to the cytosolic fraction. In addition, AXT supplementation decreased total cyclin D1 level (ELISA) compared to DMBA group (figure 2). However, no statistically significant differences were noted between hamsters fed astaxanthin alone and the control group.

**Figure 3:**
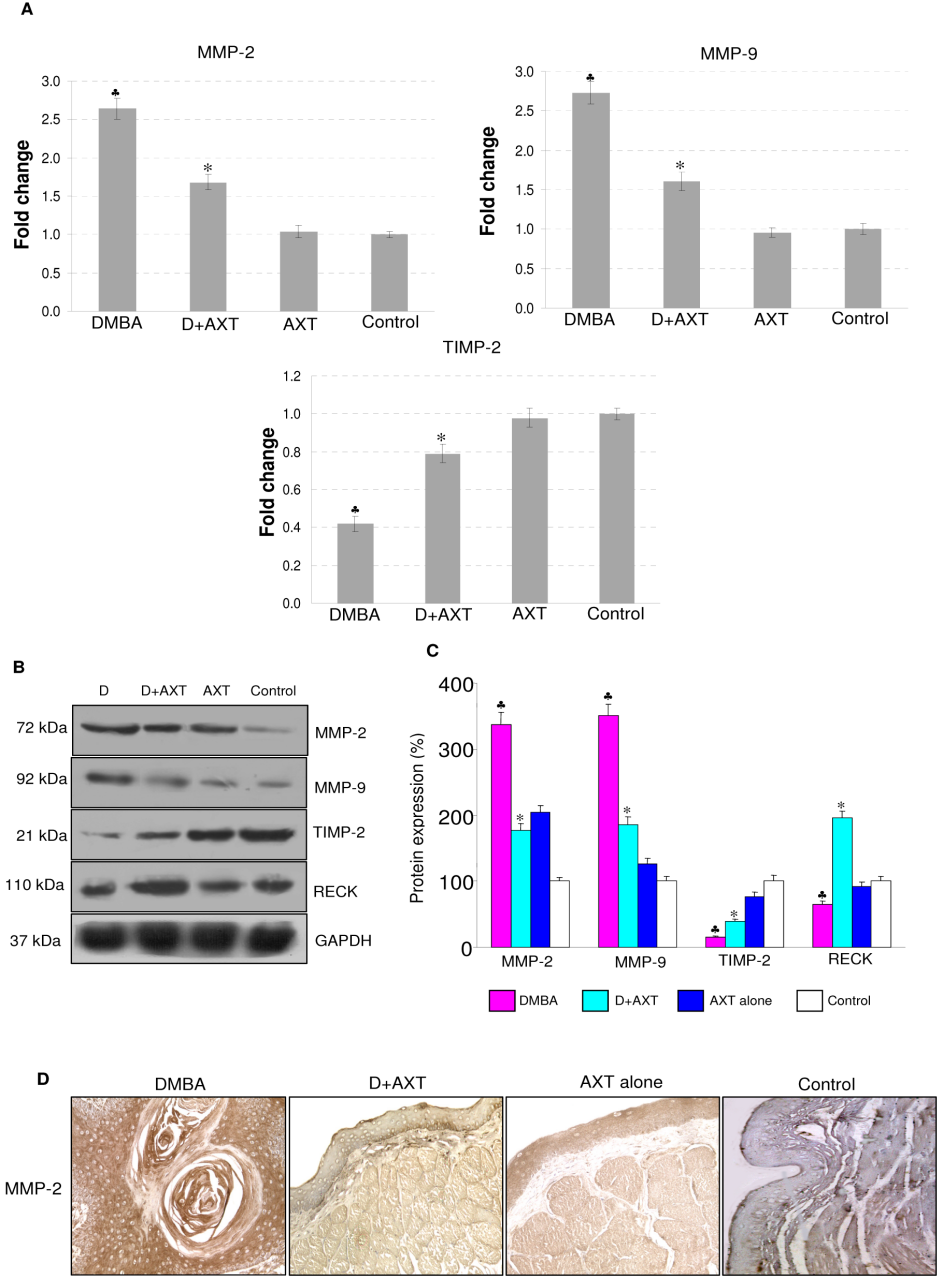
mRNA and protein expression of MMP-2, MMP-9, TIMP-2 and RECK in the buccal pouch tissues of experimental and control animals. (mean ± SD; n = 3). A. Transcript expression level of MMP-2, MMP-9 and TIMP-2 in various experimental groups as determined by kinetic PCR. Data are the mean ± SD of three independent experiments.^ ♣^p<0.05 versus control. *p<0.05 versus DMBA. **B & C**. Representative immunoblots and bar graph representing the protein expression of MMP-2, MMP-9, TIMP-2 and RECK for minimum of three independent experiments.^ ♣^p<0.05 versus control. *p<0.05 versus DMBA. D. Representative photomicrographs of immunohistochemical staining of MMP-2 in control and experimental animals (20X).

To determine whether inhibition of JAK/STAT pathway by astaxanthin impedes invasion, we next analyzed the expression of MMP-2, MMP-9 and their inhibitors. Dietary administration of astaxanthin significantly modulated the mRNA and protein expression of these molecules compared to group 1 animals. To further validate that astaxanthin regulates matrix metalloproteinases, we also examined protein expression of MMP-2 by immunohistochemical analysis. Our results revealed that astaxanthin administration markedly decreased the expression of MMP-2 compared to group 1 animals. On the other hand, no significant differences were observed in animals fed astaxanthin alone compared to control (figure 3).

**Figure 4:**
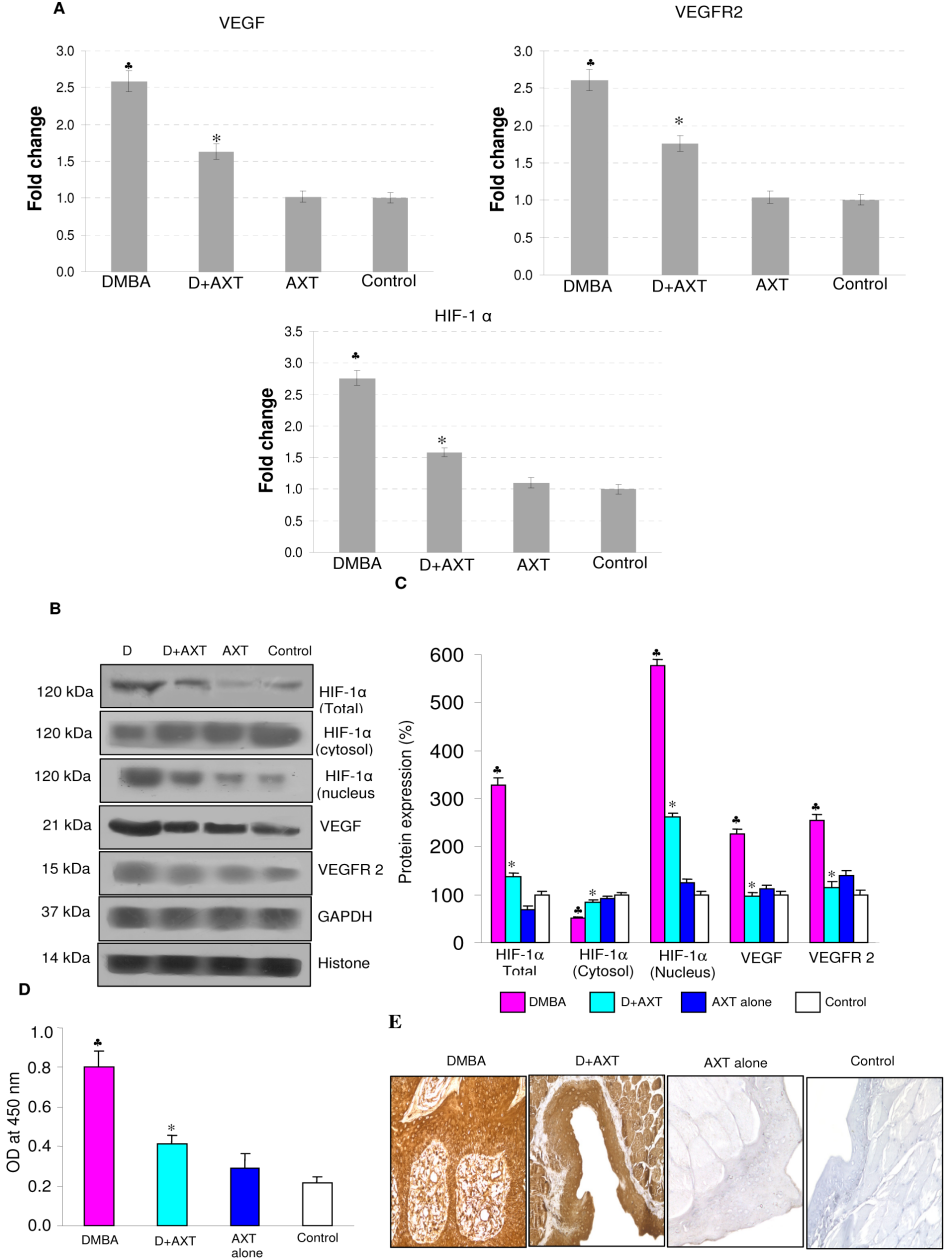
mRNA and protein expression of HIF-1α, VEGF and VEGFR2 in the buccal pouch tissues of experimental and control animals. (mean ± SD; n = 3). A. Transcript expression level of HIF-1α, VEGF and VEGFR2 in various experimental groups as determined by kinetic PCR. Data are the mean ± SD of three independent experiments.^ ♣^p<0.05 versus control. *p<0.05 versus DMBA. B. Representative immunoblot analysis. Protein samples (100 μg/lane) resolved on SDS-PAGE were probed with corresponding antibodies. GAPDH was used as loading control for cytosol and whole tissue homogenates. Histone H2B was used as loading control for nuclear proteins. C. Densitometric analysis. The protein expression from control lysates for three determinations was designated as 100% in the graph. Each bar represents the protein expression of three determinations.^ ♣^p<0.05 versus control. *p<0.05 versus DMBA. D. Levels of pVEGFR2^tyr1175^ (ELISA). E. Representative photomicrographs of immunohistochemical staining of VEGF in control and experimental animals (20X).

As neovascularisation plays a major role in tumour growth and is one of the major downstream events triggered by the JAK/STAT pathway, we investigated the effect of astaxanthin on angiogenesis. As shown in figure 4, dietary supplementation of astaxanthin significantly modulated the expression of VEGF, VEGFR2 and decreased HIF-1α nuclear translocation compared to DMBA-painted animals. Furthermore, AXT supplementation decreased the level of pVEGFR2 (ELISA). Immunohistochemical staining also confirmed decreased expression of VEGF in hamsters fed astaxanthin compared to DMBA-painted animals. No significant differences were observed in animals treated with astaxanthin alone compared to control.

**Figure 5:**
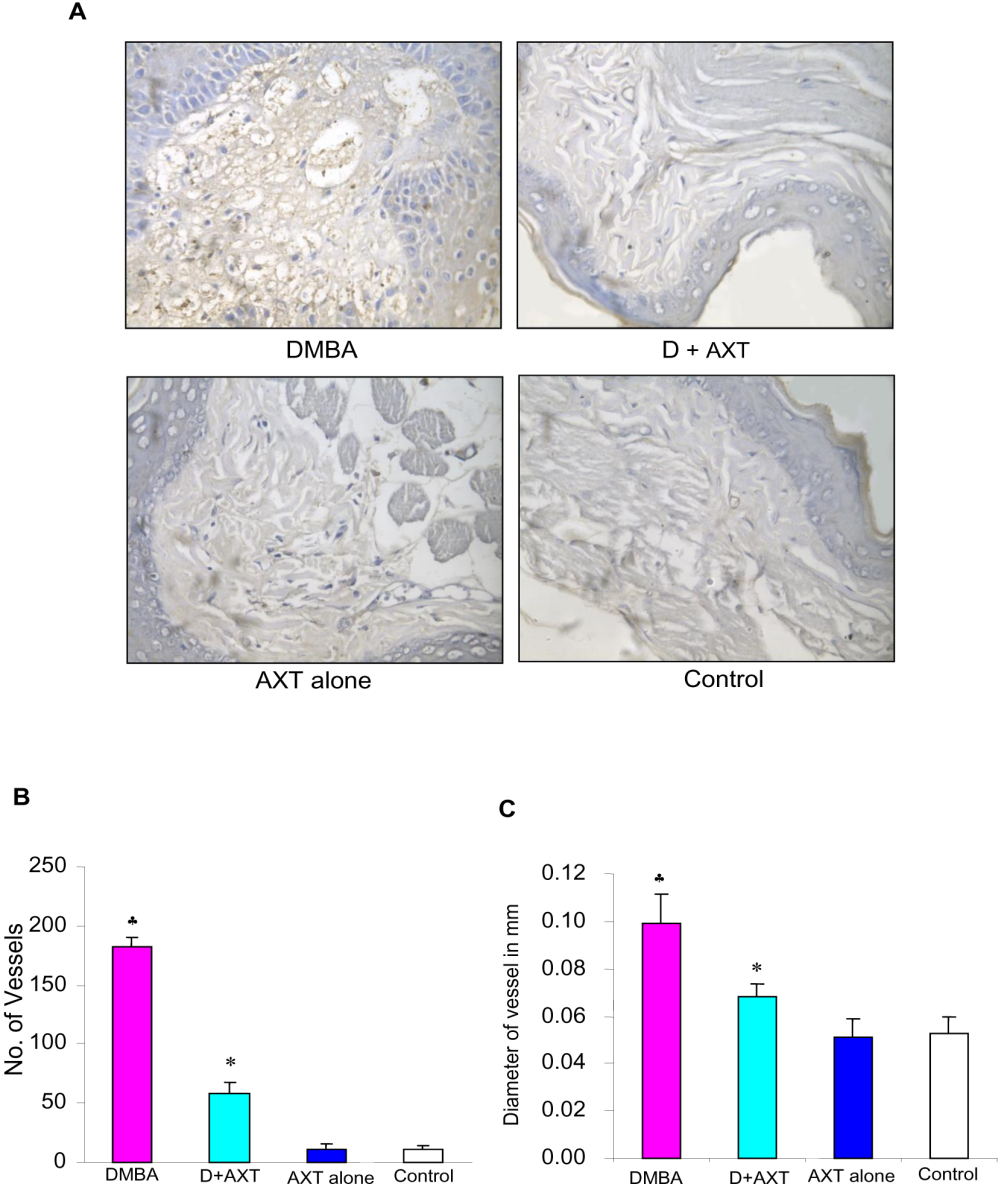
Effect of astaxanthin on microvascular density. A. Representative photomicrographs of microvascular density in control and experimental animals (40X). B. Bar graph representing number of vessels for minimum of three independent experiments.^ ♣^p<0.05 versus control. *p<0.05 versus DMBA. C. Bar graph representing vessel length for minimum of three independent experiments.^ ♣^p<0.05 versus control. p<0.05 versus DMBA.

As AXT decreased HIF-1α and VEGF expression, the key players involved in neovascularization, we next sought to determine whether inhibition of these molecules by AXT has any effect on vasculature by measuring the microvascular density. DMBA painted animals showed high vascularity with a mean MVD of 185 compared to control animals. Dietary supplementation of AXT significantly reduced the number of vessels compared to DMBA-painted animals, indicating the antiangiogenic potential of astaxanthin (figure 5).

**Figure 6:**
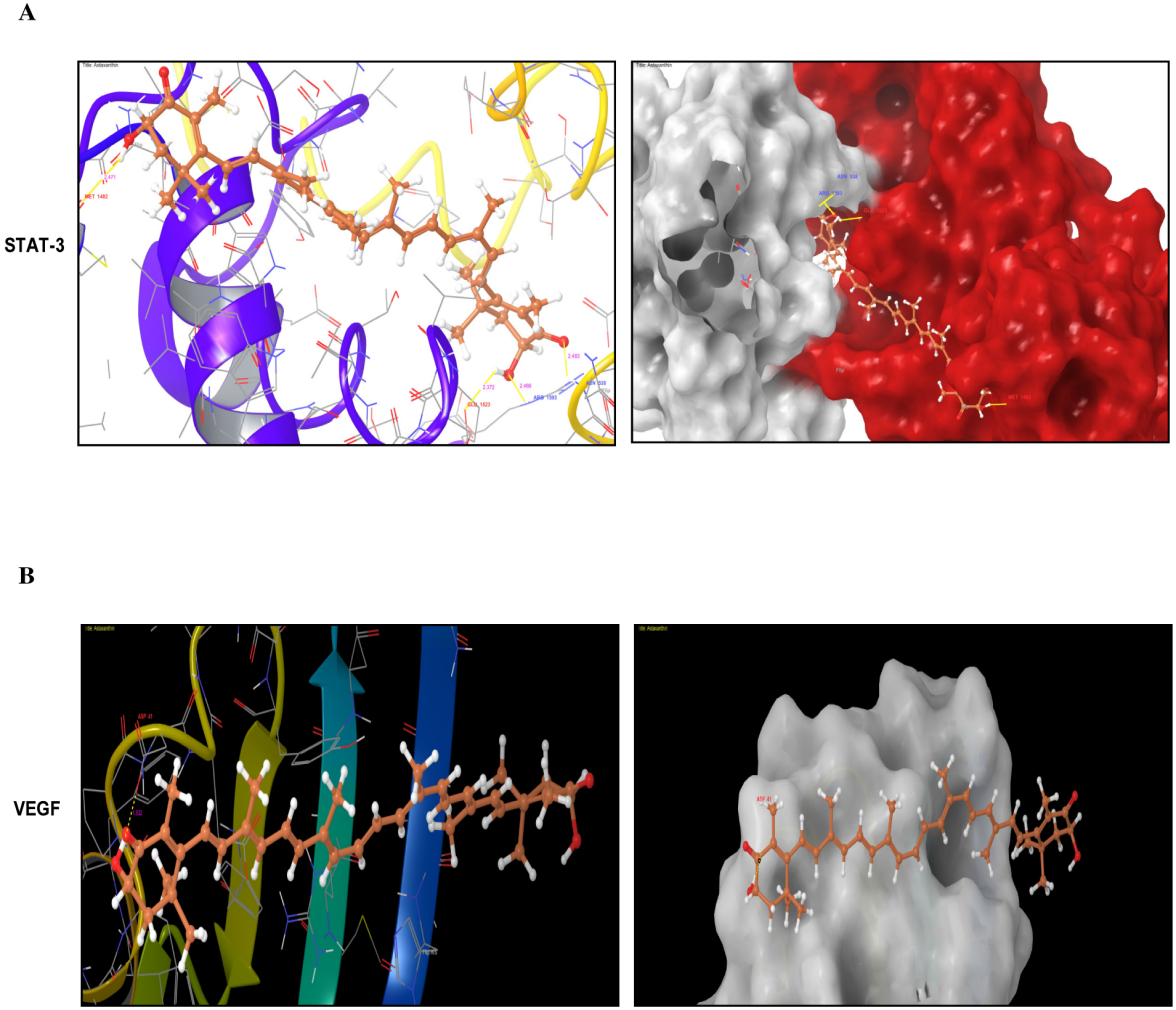
Molecular Docking. A. Represents AXT form hydrogen bond with Met 1428, Glu 1523, Arg 1593 and Asn 538 of STAT-3. B. Represents AXT form hydrogen bond with ASP 41 of VEGF.

To further confirm the inhibition of STAT-3 signaling and angiogenesis by astaxanthin, we performed molecular docking study for astaxanthin with STAT-3 and VEGF. AXT was found to bind with STAT-3 and VEGF with a docking score of −5.081 and −2.94 respectively. AXT interacts with the dimerization site of STAT-3 to form hydrogen bonds with Met 1482, Glu 1523, Arg 1593 and Asn 539 and interacts with VEGF through hydrogen bond with Asp 41 residue (Figure 6).

**Figure 7:**
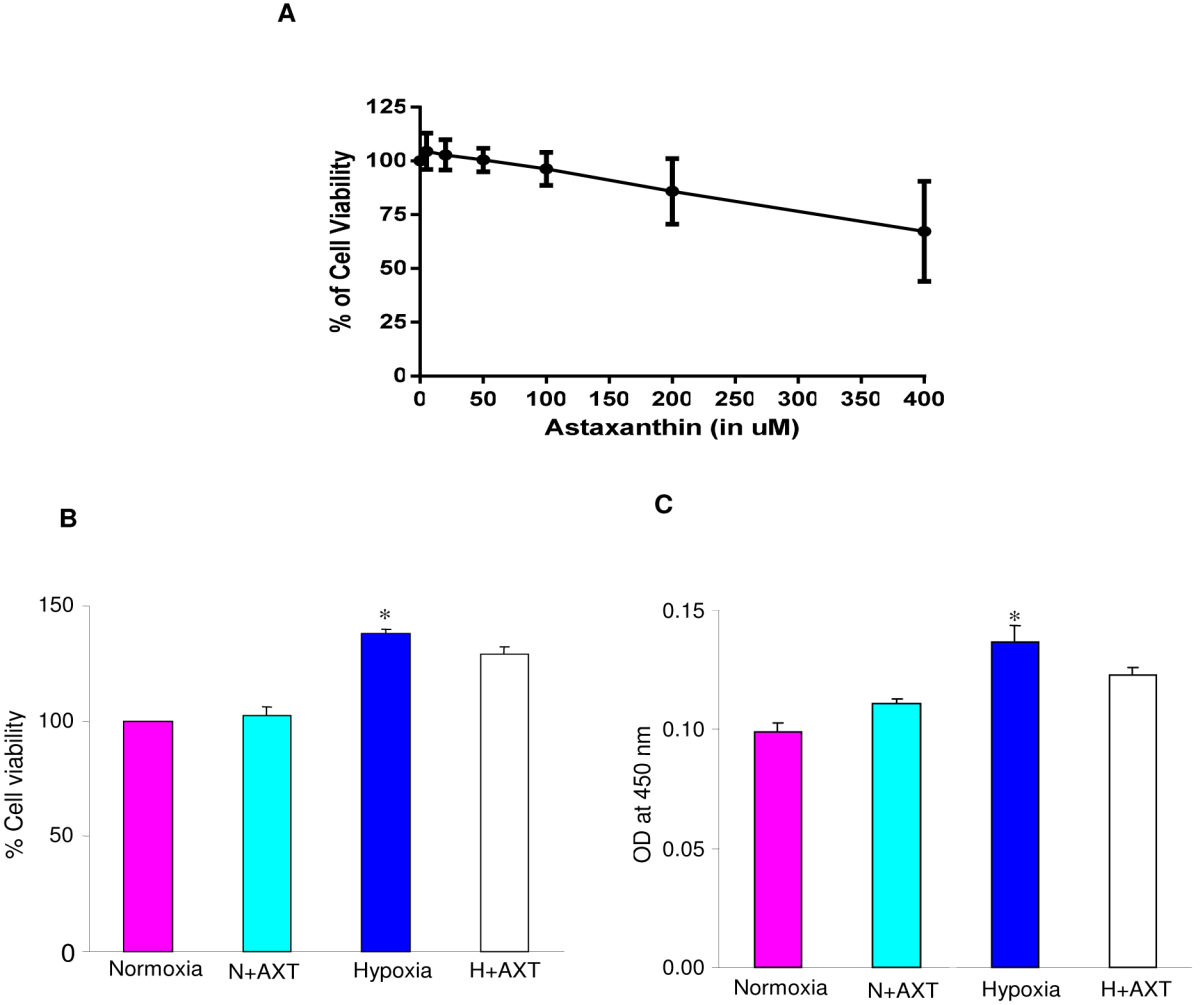
Effect of astaxanthin on cell viability and cell proliferation in ECV304 cells. **A.** IC_50_ value for astaxanthin on ECV304 cells (Alamar Blue assay). B. Effect of astaxanthin (50 µM) on hypoxia induced cell proliferation (Alamar blue assay). *p<0.05 versus normoxia. C. Effect of astaxanthin (50 µM) on hypoxia induced cell proliferation (BrdU assay). *p<0.05 versus normoxia.

To test the antiangiogenic potential of astaxanthin *in vitro* we used ECV 304 cells. Angiogenesis is a complex process that involves cell proliferation, migration, and tube formation. First, we determined the concentration of astaxanthin at which it inhibits the viability of ECV cells by 50% (IC_50_). We used varying concentrations of astaxanthin from 5 to 400 µM. We found that astaxanthin does not reduce the viability of ECV 304 cells to 50% at the concentrations tested. The viability of cells was 100% up to 50 µM of astaxanthin as shown in figure 7; therefore astaxanthin at 50 µM concentration was used for further experiments. To test whether astaxanthin inhibits proliferation of endothelial cells, we performed Alamar blue assay and BrdU assay. Hypoxia induced cell proliferation was not affected by astaxanthin as confirmed by both BrdU assay as well as Alamar blue assay (figure 7).

**Figure 8:**
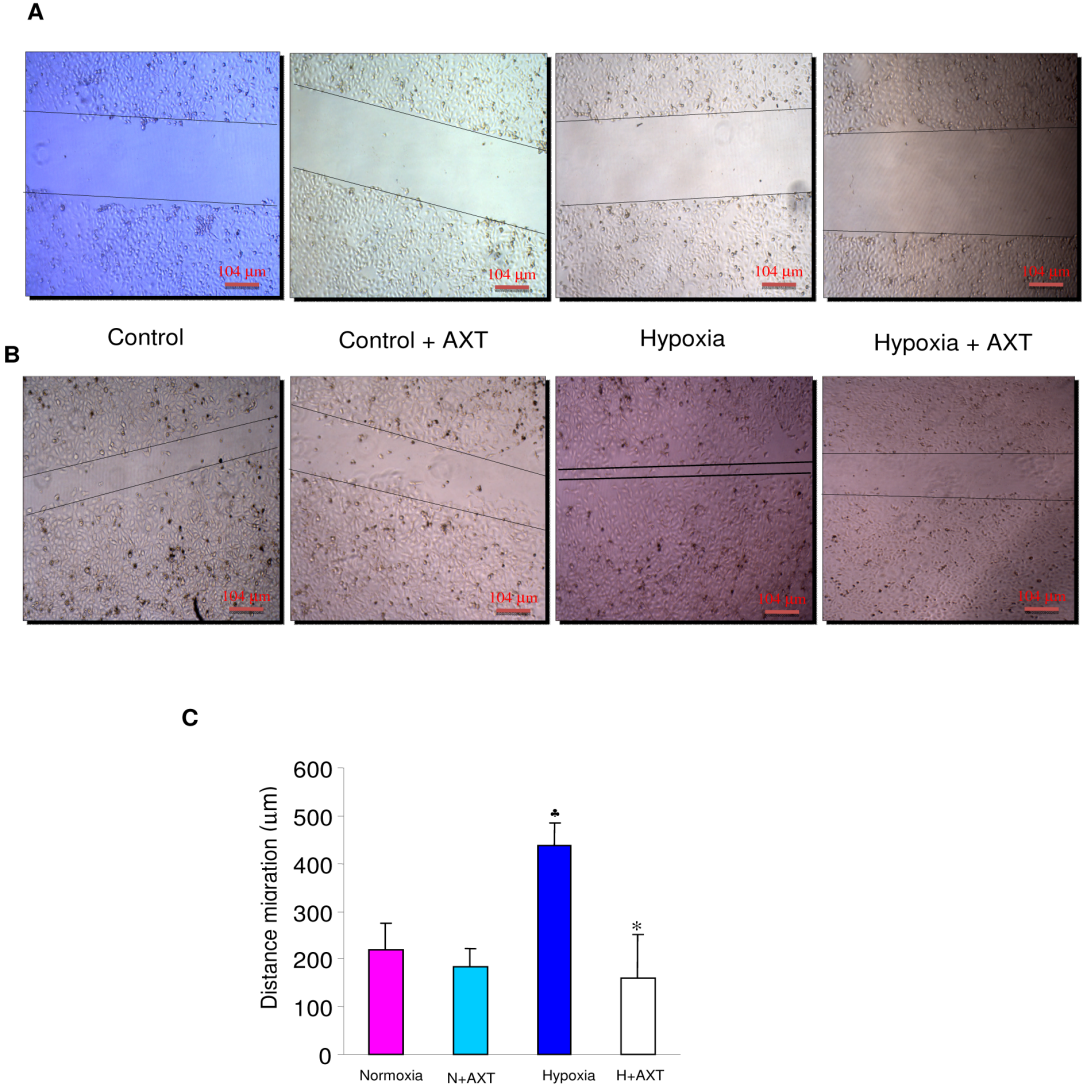
Effect of astaxanthin on the migration of ECV304 cells. A & B. Representative photomicrographs of migration assay in control and hypoxic ECV cells at 0 h and 24 h (4X). **C**. Bar graph representing distance migrated for minimum of three independent experiments.^ ♣^p<0.05 versus normoxia. p<0.05 versus hypoxia.

Migration of endothelial cells is one of the key steps in angiogenesis and metastasis; therefore we next determined the effect of astaxanthin on migration. Our results revealed that 50 µM astaxanthin significantly inhibited migration of endothelial cells. As shown in figure 8, hypoxic ECV cells migrated about 437 µm after 24 h; whereas AXT treated hypoxic cells migrated only about 192 µm, indicating the anti-migration potential of astaxanthin.

**Figure 9:**
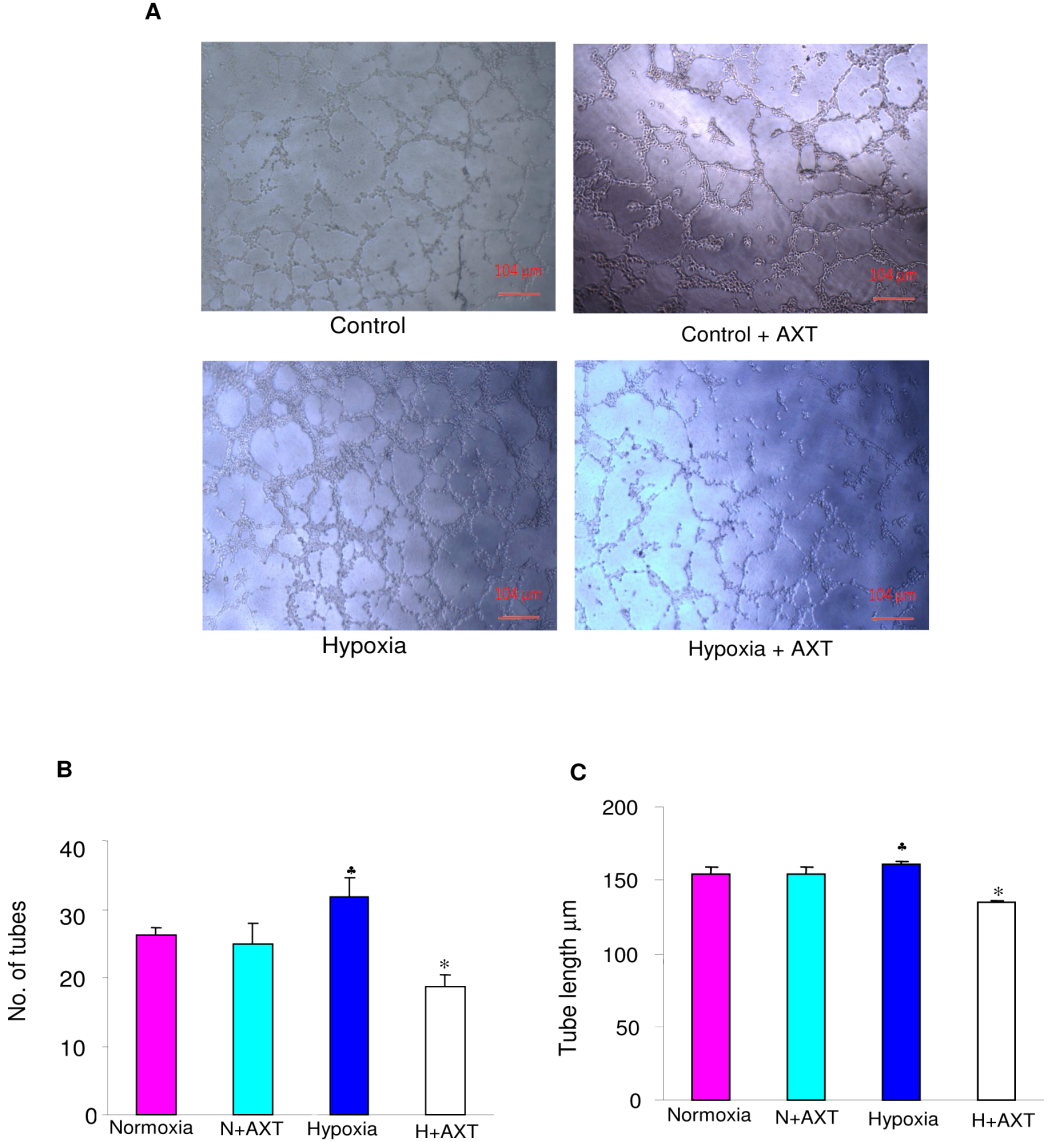
Effect of astaxanthin on tube formation in ECV304 cells. A. Representative photomicrographs of matrigel tube formation assay in control and hypoxic ECV cells (4X). B. Bar graph representing no. of tubes for minimum of three independent experiments.^ ♣^p<0.05 versus normoxia. *p<0.05 versus hypoxia. C. Bar graph representing tube length for minimum of three independent experiments.^ ♣^p<0.05 versus normoxia. *p<0.05 versus hypoxia.

The principal step during angiogenesis is the formation and merging of tubes produced by endothelial cells forming a complex network of vessels, hence we next determined the effect of AXT on tube formation by performing matrigel tube formation assay. Under hypoxic condition, there was significant increase in the number of tubes formed as well as tube length compared to normoxic condition. Astaxanthin treatment significantly reduced the number of tubes and tube length under hypoxic condition. No significant differences were observed in cells treated with astaxanthin under normoxic condition (figure 9).

**Figure 10:**
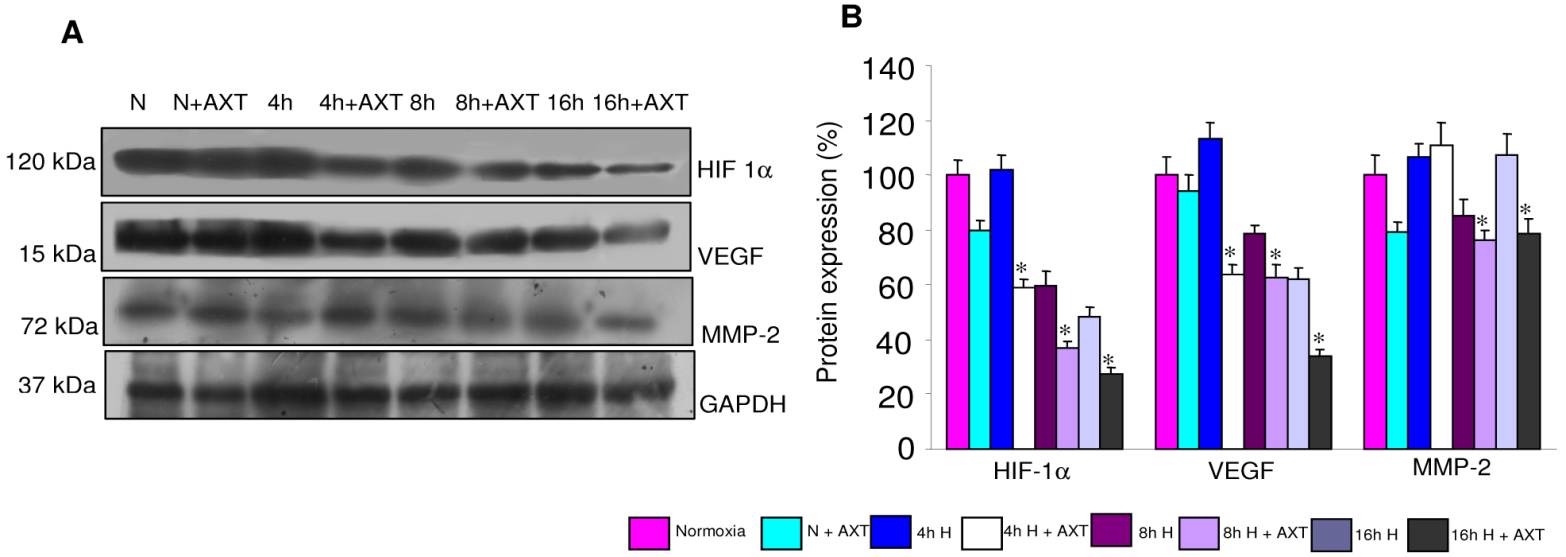
Effect of astaxanthin on the expression of HIF-1α, VEGF, and MMP-2 in normoxic and hypoxic ECV304 cells. **A & B.** Representative immunoblots and bar graph representing protein expression of HIF-1α, VEGF and MMP-2 for minimum of three independent experiments. *p<0.05 versus hypoxia.

To know the mechanism by which AXT inhibits migration and tube formation of endothelial cells, we analyzed the expression of pro-angiogenic molecules in normoxic and 4 h, 8 h, and 16 h hypoxic ECV cells. Immunoblot analysis revealed an increase in HIF1α expression from 4 h that was sustained up to 8 h hypoxia. VEGF and MMP-2 expression increased from 8 h onwards and persisted till 16 h. Treatment with AXT reduced hypoxia-induced increase in the expression of these molecules (Figure 10).

## Discussion

Several studies have documented that aberrant activation of the STAT-3 signaling pathway contributes to neoplastic transformation in various malignancies, and have validated STAT-3 as a promising target for cancer therapy [11], [30], [31]. The development of agents that target STAT-3 with adequate potency and tumour selectivity has proven to be a difficult task. Studies by others and us have indicated that phytochemicals are involved in cancer chemoprevention by modulating the signaling circuits aberrant in cancer [32]–[35]. In the present study, we demonstrate that astaxanthin inhibits the JAK/STAT-3 signaling pathway as well as its target genes in the HBP carcinoma model.

The functions of STAT-3 protein mainly depend on its phosphorylation and subcellular localization. In unstimulated cells, the STAT-3 proteins are present in the inactive form in the cytosol. Activation of STAT-3 occurs through phosphorylation of its tyrosine residue by cytokine or growth factor receptor signaling. Phosphorylated STAT-3 then dimerizes and translocates to the nucleus where it binds to IFN-gamma-activated site (GAS) in DNA and activates the transcription of target genes [12]. STAT-3 is found to be constitutively active in different carcinomas and inhibition of STAT-3 activation correlates with suppression of malignant cells both *in vitro* and *in vivo* [36], [37]. The results of the present study provide evidence that astaxanthin supplementation abrogates constitutive activation of STAT-3 by preventing its phosphorylation and subsequent nuclear translocation. Furthermore, the interaction of AXT with the dimerization site of STAT-3 by molecular docking studies also validates the inhibition of STAT-3 by AXT. Astaxanthin was shown to inhibit rat hepatocellular carcinoma CBRH-7919 cells by modulating the JAK/STAT-3 signaling [38]. Taken together, these studies demonstrate that astaxanthin reduces the nuclear pool of STAT3, a key event in JAK/STAT-3 signaling.

Accumulating evidences have shown a positive correlation between the inhibition of STAT-3 signaling and suppression of tumor cell proliferation. Downregulation of the cell cycle regulatory proteins cyclin D1 and PCNA, biomarkers of the malignant phenotype and tumor progression with increased expression of p21 the most potent CDK inhibitor, underscore the antiproliferative effects of astaxanthin. These findings are substantiated by those of Zhang et al. [39] who demonstrated that astaxanthin inhibits cell proliferation in K562 cancer cells by modulating the expressions of cyclin D1 and p21. While targeting cyclin D1 can have far reaching implications in inhibiting cell proliferation, invasion, and angiogenesis, enforced nuclear localization of p21 seen in the present study can arrest the cell cycle by binding to PCNA [40], [41].

Alterations in the levels and distribution of proteins involved in invasion have been documented in the HBP model [42]. Both STAT-3 and cyclin D1 are known to influence the expression of genes that promote migration and invasion [9], [40]. Our results reveal that dietary astaxanthin significantly reduced the expression of matrix metalloproteinases (MMPs), MMP-2 and MMP-9, the crucial players involved in the degradation of the extracellular matrix and upregulated the expression of negative regulators of MMPs, TIMP-2 and RECK. In line with our findings, astaxanthin was shown to inhibit dimethylhydrazine induced rat colon carcinogenesis by modulating the expressions of MMPs, NF-κB and Erk [43].

Aberrant activation of STAT-3 signaling is also recognized to stimulate angiogenesis by activating VEGF, a pro-angiogenic molecule via direct binding with its promoter [44]. In the present study, abrogation of STAT-3 signaling by astaxanthin was found to be associated with downregulation of the key mediators of angiogenesis VEGF and VEGFR2. Interestingly, astaxanthin blocked nuclear translocation of HIF-1α, a master regulator of angiogenesis that transactivates several hypoxia responsive genes including VEGF and its receptors. The significant reduction in MVD in astaxanthin supplemented hamsters confirms its anti-angiogenic potential. Astaxanthin was also reported to inhibit the development of experimental choroidal neovascularization by modulating the expression of VEGF and VEGFR2 [45].

In tumours, hypoxia stimulates accumulation of HIF-1α by inhibiting proteasomal degradation which in turn stimulates angiogenesis by upregulating the expression of MMP-2 and VEGF [46], [47]. Several phytochemicals have been reported to inhibit hypoxia induced angiogenesis by modulating the expression of pro-angiogenic molecules [48], [49]. In the present study, astaxanthin inhibited hypoxia induced migration and tube formation of ECV 304 cells but did not significantly alter hypoxia induced cell proliferation. Immunoblotting analysis revealed that astaxanthin inhibited hypoxia induced angiogenesis by significantly decreasing the expression of HIF-1α, VEGF and MMP-2. Inhibition of angiogenesis was further supported by molecular docking studies that showed interaction of AXT with VEGF.

## Conclusion

**Figure 11:**
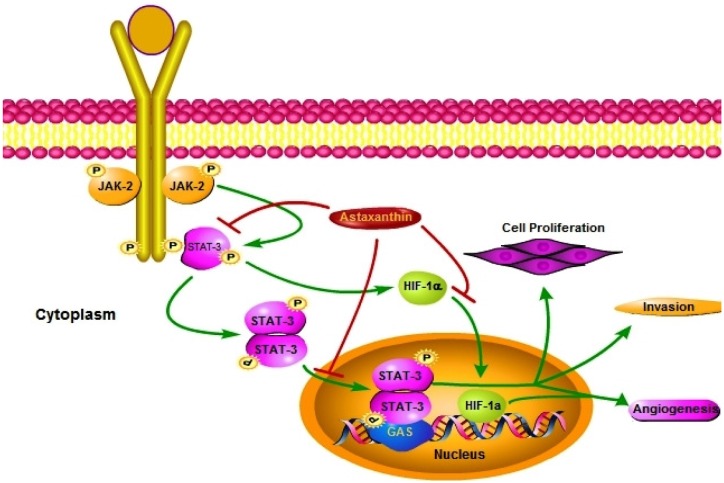
Schematic representation of the mechanism of action of astaxanthin in DMBA induced oral cancer. Dietary supplementation of astaxanthin abrogates DMBA induced oral cancer by targeting JAK/STAT-3 signaling. Astaxanthin prevents the phosphorylation and nuclear translocation of STAT-3 thereby prevents the transactivation of STAT-3 target genes which are involved in cell proliferation, invasion and angiogenesis. Furthermore astaxanthin also prevents nuclear translocation of HIF-1α, the master regulator of angiogenesis.

In summary, the results of the present study provide substantial evidence that astaxanthin inhibits DMBA induced HBP carcinomas by attenuating JAK/STAT signaling (Figure 11). Astaxanthin appears to reduce the aberrant activation of STAT3 by various strategies including suppression of pSTAT3tyr705, blocking nuclear translocation of the active dimer and associated downstream signaling, and preventing transactivation of STAT3 target genes that play pivotal roles in cell proliferation, invasion, and angiogenesis. In addition, astaxanthin also inhibited endothelial cell migration and tube formation by modulating pro-angiogenic molecules. Our study supports the widely held tenet that inhibition of STAT-3 activation is an attractive strategy for modulating the expression of genes implicated in tumour development and progression. Dietary phytochemicals such as astaxanthin that function as potent inhibitors of JAK/STAT signaling are promising candidate agents for cancer chemoprevention and anticancer therapeutics.
